# Novel *PTCH1* Mutations in Patients with Keratocystic Odontogenic Tumors Screened for Nevoid Basal Cell Carcinoma (NBCC) Syndrome

**DOI:** 10.1371/journal.pone.0043827

**Published:** 2012-08-27

**Authors:** Lorenza Pastorino, Annamaria Pollio, Giovanni Pellacani, Carmelo Guarneri, Paola Ghiorzo, Caterina Longo, William Bruno, Francesca Giusti, Sara Bassoli, Giovanna Bianchi-Scarrà, Cristel Ruini, Stefania Seidenari, Aldo Tomasi, Giovanni Ponti

**Affiliations:** 1 Department of Internal Medicine and Medical Specialties (DiMI) University of Genoa, Genoa, Italy; 2 Oral Medicine Unit, Department of Odontostomatological and Maxillofacial Sciences, School of Medicine and Surgery, Federico II University of Naples, Naples, Italy; 3 Division of Dermatology, Department of Head and Neck Surgery, University of Modena and Reggio Emilia, Modena, Italy; 4 Dermatology Unit, 1st Medical Department, Arcispedale Santa Maria Nuova, Reggio Emilia, Reggio Emilia, Italy; 5 Laboratory of Genetics of Rare Hereditary Cancers, IRCCS AOU San Martino-IST, Genoa, Italy; 6 Department of Clinical and Diagnostic Medicine and Public Health, University Hospital of Modena and Reggio Emilia, University of Modena and Reggio Emilia, Modena, Italy; Southern Illinois University School of Medicine, United States of America

## Abstract

Keratocystic odontogenic tumors (KCOTs) are cystic tumors that arise sporadically or associated with nevoid basal cell carcinoma syndrome (NBCCS). NBCCS is a rare autosomal dominantly inherited disease mainly characterized by multiple basal cell carcinomas, KCOTs of the jaws and a variety of other tumors. *PTCH1* mutation can be found both in sporadic or NBCCS associated KCOTs. The aim of the current study was to assess whether a combined clinical and bio-molecular approach could be suitable for the detection of NBCCS among patients with a diagnosis of keratocystic odontogenic tumors (KCOTs). The authors collected keratocystic odontogenic tumors recorded in the database of the Pathology Department of the University of Modena and Reggio Emilia during the period 1991–2011. Through interviews and examinations, family pedigrees were drawn for all patients affected by these odontogenic lesions. We found out that 18 of the 70 patients with KCOTs and/or multiple basal cell carcinomas actually met the clinical criteria for the diagnosis of NBCCS. A wide inter- and intra-familial phenotypic variability was evident in the families. Ameloblastomas (AMLs) were reported in two probands that are also carriers of the *PCTH1* germline mutations. Nine germline mutations in the *PTCH1* gene, 5 of them novel, were evident in 14 tested probands. The clinical evaluation of the keratocystic odontogenic tumors can be used as screening for the detection of families at risk of NBCCS. Keratocystic odontogenic lesions are uncommon, and their discovery deserves the search for associated cutaneous basal cell carcinomas and other benign and malignant tumors related to NBCCS.

## Introduction

The odontogenic keratocyst is a cystic lesion that has a putative growth potential and a propensity for recurrence [1], [2]. Although the great majority of keratocysts occur in isolation as single, non-syndromic cysts, they may also present as multiple cysts as a feature of the nevoid basal cell carcinoma syndrome [NBCCS or Gorlin syndrome-GS, OMIM#109400]. NBCCS is a rare autosomal dominantly inherited disorder with variable clinical manifestations, such as basal cell carcinomas of the skin, keratocysts of the jaws, palmar or plantar pits, ectopic calcifications of the falx cerebri [3], [4]. The estimated birth incidence of the disorder is approximately 1 per 31,000, ranging from 1 per 19,000 in the United Kingdom to 1 per 256,000 in Italy to 1 per 235,800 in Japan [5]–[7].

Multiple jaw keratocysts are the most consistent and common manifestation of the syndrome, occurring in 65–100% of patients [3]. The syndrome-associated keratocysts are found in both jaws with equal frequency, in contrast to non-syndromic cysts, which are most frequently associated with the lower jaw [8]. Keratocysts often represent the first manifestations of NBCCS, frequently preceding syndromic basal cell carcinomas, thus facilitating early diagnosis [9].

In 2005, the WHO working Group recognized Odontogenic keratocysts as tumors and recommended the use of the term keratocystic odontogenic tumor (KCOT), in order to distinguish the lesion from the ortho-keratinizing variant, which is considered as an odontogenic cyst [10]. The histologic diagnosis of KCOTs is primarily based on the presence of specific microscopic features: a thin, stratified squamous epithelium with a prominent palisaded basal layer; a smooth interface with the stroma, lacking rete pegs and a wavy or corrugated parakeratinized surface layer. Immunohistochemical staining for keratin 10, a low-molecular-weight cytokeratin that is expressed in a subset of keratinocytes within normal gingival mucosa, shows a characteristic strong staining of the superficial parakeratinized cells of KCOTs and can be applied to both histologic and cytological samples as an ancillary marker to support the diagnosis of an KCOT [11]–[13].

*PTCH1* mutation can be found in sporadic or NBCCS associated KCOTs [8]. The PTCH protein serves as a receptor for the Secreted Shh (SHH) protein, and inhibits the signaling pathway by repressing the activity of Smoothened (SMO), another transmembranous protein [14]. The SHH signaling pathway plays an important role in mammalian embryonic development of structures such as the neural tube, axial skeleton, limbs, lungs, skin, hair follicles, and teeth [15]. SHH signaling also regulates growth and determines the shape of teeth. [16].

The specific objective of our study was to evaluate whether a matched clinical and molecular screening could be useful for the recognition of NBCCS among patients with a diagnosis of KCOTs.

## Results

Among 70 patients affected by KCOTs there were 40 males and 30 females (ratio 1∶1.3 ). Their age ranged from 14 to 86 years (mean 50.8).

We collected clinical characterization and family history of all patients. 18 patients had a personal and/or family history of BCCs and/or other signs and symptoms fulfilling the criteria for NBCCS. Among these NBCCS probands there were 9 males and 9 females. The male to female ratio of the NBCCS probands was 1∶1.

**Figure 1:**
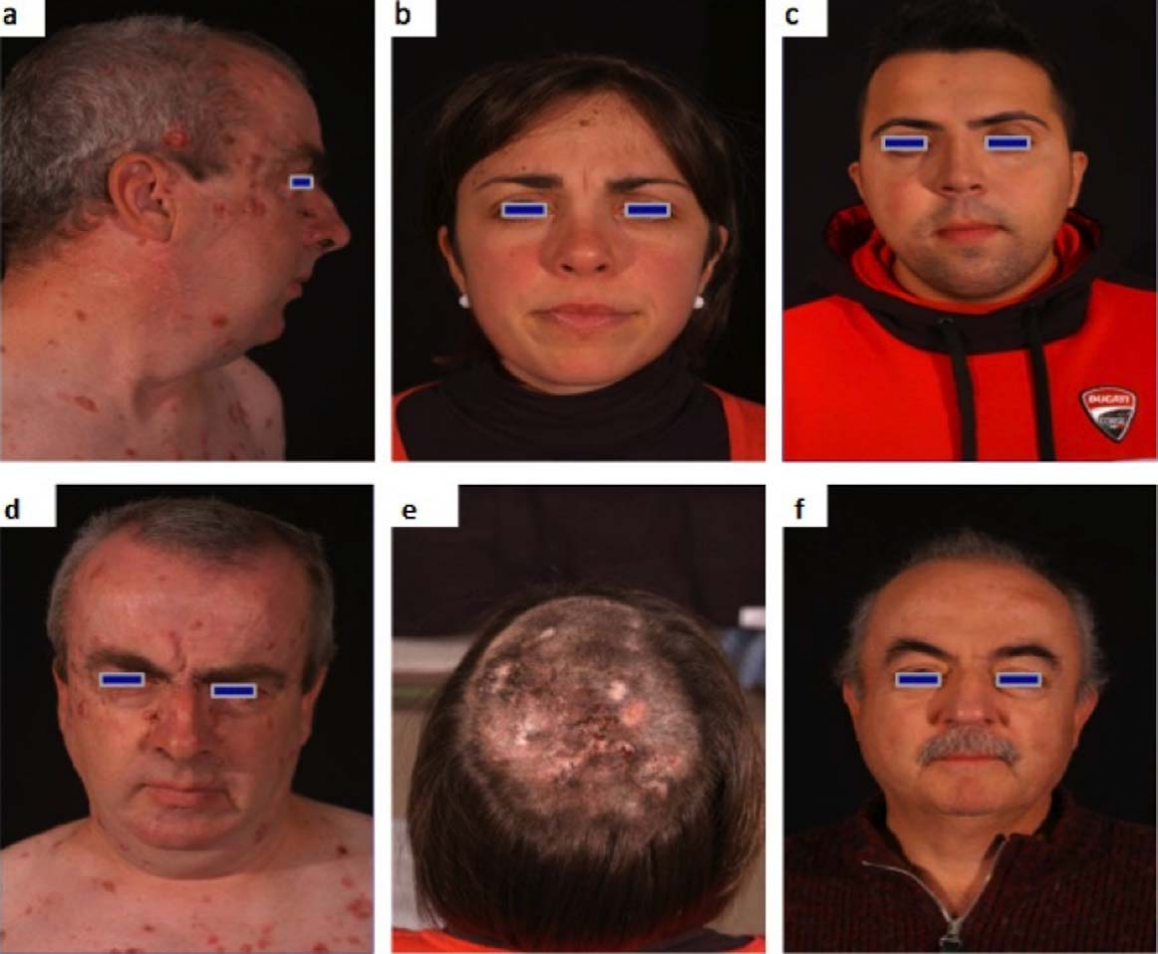
Clinical features of NBCCS’ patients (a, d : patient n. 3; b, e : patient n.16; c: n.11 proband’s’brother; d: n. 11 proband’s father; see **Table 2**).

BCCs were preferentially located on the scalp in females and on the lower limbs in males. The face was equally affected in males and female whereas the trunk was predominantly involved in males, especially in the lower portion; instead, females showed the prevalence of BCCs in the upper trunk [Figure 1].

All of the remaining 52 patients were categorized with sporadic KCOTs tumors. In the NBCCS group, there were approximately 55 KCOTs, including 2 AMLs. The average age at onset of the first KCOT was 18,8 years compared with 45,5 years in the sporadic group [Table 1].

**Table 1:** Odontogenic tumors in patients with Nevoid Basal cell Carcinoma Syndrom and in patients with sporadic lesions.

	NBCCS patients	Non syndromic patients	P Valueα<0.05
N patients	18 (25.7%)	52 (74.3%)	–
**Gender:**			
Female	9 (12.8%)	21 (30%)	–
Male	9 (12.8%)	31 (44.4%)	–
**KCOTs:**			
KCOTs per patient (mean ± SD)	2.9±2.5	1.4±0.75	0.0001
KCOTs onset yrs (mean ± SD)	18.8±9.2	45.5±16.9	0.0014
**KCOTs location:**			
Upper jaw	18 (34.6%)	16 (22%)	–
Lower jaw	34 (65.4%)	57 (78%)	–
**Histology:**			
KCOTs	52 (41.2%)	73 (58,8%)	–
AML	2 (100%)	0 (0%)	–

KCOTs: Keratocistic odontogenic tumors;

AML: ameloblastomas;

SD: Standar.

**Figure 2:**
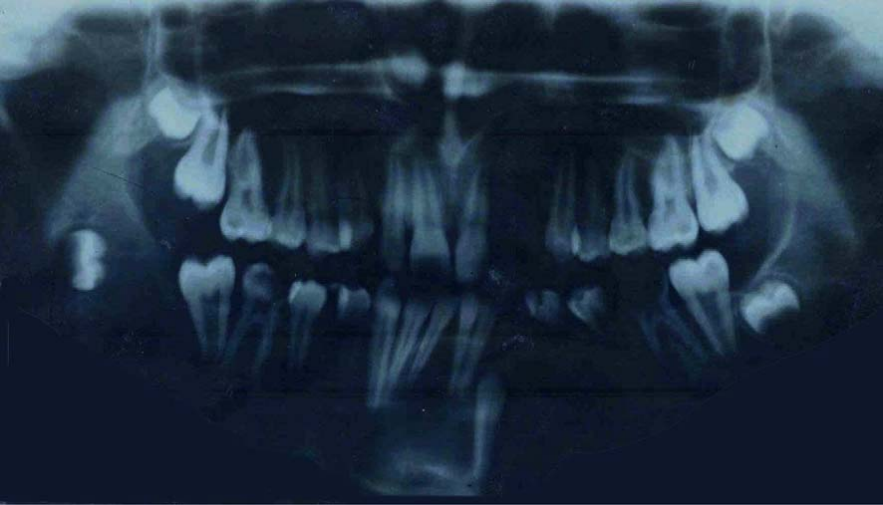
Dental Orthopantomography of a patient affected by NBCCS. (Patient n.11 of **table 2**).

The great majority of cysts arise sporadically and in single form on jaws of middle-aged people. When associated with the NBCCS, these lesions appear earlier, often during the first or second decade of life, and they are often multiple, synchronous or metachronous [Figure 2] [Table 2].

**Table 2:** Features of Nevoid Basal Cell Carcinoma Syndrom patients.

Pt	Age	Sex	Number of KCOTs	KCOTs onset (yrs)	Number of BCCs	BCCs onset (yrs)	BCCs localisation	Patients’ gene mutations	EX/IN	Underlying diseases	Deceased	Relatives Affected	Relatives’ KCOTs/BCCs	Relatives’ gene mutations
1	33	F	2	14	>10	25	Forehead, upper lip, shoulder	c.3277G>A G1093R	Ex19	Palmo-plantar pits	No	Father	BCCs	
2	39	M	6	35	>10	30	Upper chest, back	c.1987C>T [p.Q663X]	Ex 14	Thymoma, Chest cyst	No	No		
3	46	M	2	11	>10	18	Face, trunk	c.654+2T>A	IN 5	Hydrocefalus, Polydactylism left foot, Bifid rib	No	Father	BCCs KCOTs	
4	53	M	2	16	>10	35	Face, trunk, Upper legs	c.2062C >T [p.Q688X]	Ex 14	Bifid rib	No	Daughter	Polydactylism right foot Bifid rib	
5	28	M	2	13	>7	18	Lumbosacral, scalp			Fibroepithelioma of Pinkus	No	Father	NBCCs	
6	30	F	1	30	1	30					No	No		
7	62	M	5	18	>10	30	Total body (from scalp to ankle)			Kaposi’s sarcoma	No			
8	40	M	10	11	>7	32		c.2186A>T [p.K729M]	Ex 14	Skeletal anomalies, AML	No	Son	Frontal bossing NBCCs	c.2186A>T [p.K729M]
9	43	M	1	12	>7	38	Nasal–labial sulcus, retroauricular, Shoulder, neck, clavicle			Strabismus	No	No		
10	82	M	1	35							Yes			
11	22	F	5	13	NO			C.1348-2A>G	IN 9	Epidermoid cyst, Cafe au lait spots	No	Father Brother	KCOTs, frontal bossing	C.1348-2A>G
12	40	F	3	28	>7			c.585-1G>A	IN 3		No	Father Sister Daughter	Deceased (KCOTs and BCCs) (KCOTs and BCCs) 350 BCCs, fetal thoracic rhabdomyoma	c.585-1G>A
13	46	F	1	29	10	38	Nose, scalp, back				No	Mother	BCCs	
14	61	F	1	15	5	35	Nose, cheeks, neck	c.931insA	EX 6	Ovarian fibromas, AML	No	Father	KCOTs	
15	64	M	6	13	>10	16	Face, neck, trunk, arms	c.1237C>T [p.Q413X]	EX 9	Palmo-plantar pits, Macrocephaly	No			
16	33	F	3	15	30	11	Scalp, shoulder			Macrocephaly, Hypertelorism	No			
17	59	F	1	4	7	20	Face, trunk, arm			Uterine fibroma	No	Brother	BCCs	
18	55	F	1	26	10	43				Uterine fibroma	No			

Abbreviation: BCC = BASAL CELL CARCINOMA KCOT = KERATOCYSTIC ODONTOGENIC TUMOR.

In 10 patients with NBCCS, KCOTs occurred as the first neoplasm, while they were diagnosed in 8 patients only after the development of BCCs.

**Figure 3:**
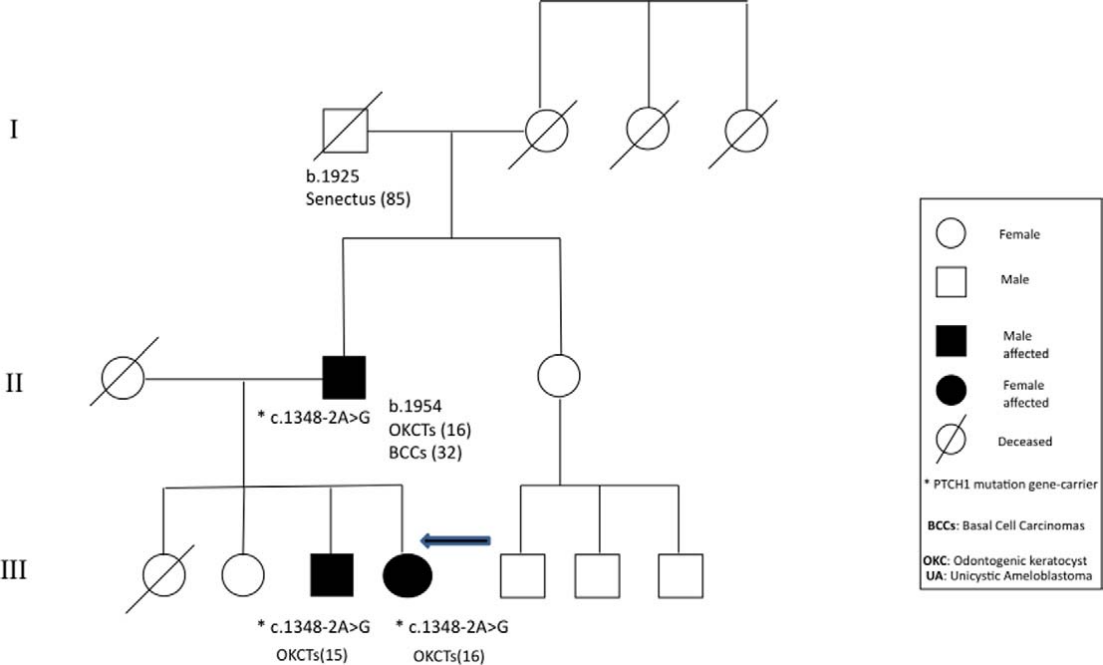
Genealogic tree of NBCCs family without the diagnosis of BCCs (Patient n.11 of **table 2**).

Among the 16 NBCCS probands with diagnosis of basocellular carcinomas, the number of BCCs varied from a few (patient number 17) to several thousands (patient number 3) and ranged in size from 1 to 35 mm in diameter. The average age at onset of the first BCC was 27,5 years. The remaining one did not show any BCCs [Figure3].

Although the histopathology of BCCs in NBCCS patients cannot be differentiated from that of sporadic BCCs, approximately 30% of patients present two or more histologic sub-types of BCCs with superficial, nodular, cystic, sclerosing patterns and fibroepithelioma of Pinkus (patient number 5) [Table 2].

Overall, a wide phenotypic and genotypic variability was registered, in the spectrum of both skin and odontogenic tumors, even among members of the same family who had different clinical manifestations.

In addition to odontogenic and cutaneous tumors, the tumor spectrum included thymoma, fetal thoracic rhabdomyoma, giant cells tumors and ovarian fibroma.

The relationship between the development of KCOTs and other main features of the syndrome (BCCs, brain tumors, palmar/plantar pits, abnormal ribs, abnormal vertebrae, heart tumors, and ovarian tumors) was also examined. Probands with NBCCS who had been affected by KCOTs had a median of three other features of the syndrome. The most common features were BCCs (95%), abnormal ribs and vertebrae (23%) and palmar/plantar pits (11%). Sporadic patients with KCOTs were also screened for clinical features (i.e. other neoplasms) that are not associated to NBCCS in order to analyze their potential associations.

The results of the molecular analysis of 14 consenting Italian patients with histologically confirmed NBCCS are shown in Table 2. We found 9 germline *PTCH1* mutations (60%) which appeared to be uniformly distributed across the *PTCH1* gene. Seven of them (77,8%) belonged to patients with family history of NBCCS. To our knowledge, 5 of them (c.654+2T>A, c.1348-2a>G, c.585-1G>A, c.931dupA, c.1237C>T [p.Q413X] were novel and had not been described yet, 2 (c.3277G>A [p.G1093R], c.2186A>T [p.K729M]) had been recently reported [17], [18] and 2 had already been reported in the literature (c.1987C>T [p.Q663X], c.2062 C>T [p.Q688X]) [19].

## Discussion

A combined clinical and molecular screening could be useful for the recognition of NBCCS in patients that already have an early diagnosis of multiple KCOTs, starting with the evaluation of retrospective pathological records. NBCCS is usually diagnosed through the detection of multiple BCCs at the skin examination; our approach chose to guide the discovery of affected patients starting from another major criteri (KCOTs). The majority of the KCOTs in this series (74,8%) were sporadic and not associated with the mutation of *PTCH1*. These results clearly indicate that most KCOTs develop as sporadic forms, confirming the discriminatory role of an accurate family medical history that should be viewed as the crucial step for a proper diagnosis of NBCCS. In particular, the case-history can be very helpful in the diagnosis of the syndrome in young children that only present KCOTs, but its role is limited to NBCCS patients who are the first affected in their families. The early recognition of *PTCH1* gene carriers allows the assessment of a strict clinical and instrumental follow-up, leading to the detection of further tumors such as other BCCs and KCOTs, as well as medulloblastomas in affected children,

In this study, approximately 25,6% of KCOTs patients were associated with the autosomal dominantly inherited NBCCS. Of these patients, 11 presented multiple KCOTs. In a previous study the diagnosis of NBCCS was associated with 6% of all KCOTs, percentage similar to those of other reports, which ranged between 1,4% and 8,2% [20]–[23]. The higher incidence of NBCCS in patients with odontogenic cysts can be reasonably explained by two factors. Firstly, in our study, an accurate family history (including a detailed recording of radiographic and photographic pictures), was extended to second, third and fourth degree relatives (instead of being limited to second-degree relatives). Secondly, all patients were accurately screened for basal cell carcinomas with dermoscopy. Dermoscopy is an imaging technique that has been proven to greatly improve the diagnostic accuracy of BCCs and other skin tumors compared to naked eye examination. Together, these aspects can support the higher incidence of NBCCS in our patients, since previous studies did not explore the entire family pedigree of the probands; moreover, the diagnosis of BCC was performed through the clinical examination alone.

BCCs are not constantly associated to KCOTs in patients with *PTCH1* mutation. Normally, the only sign of BCCs not associated to KCOTs is not enough to allow the suspect of NBCCs, although some patients develop the other requested signs for the clinical diagnosis of NBCCS (according to Kimonis’s criteria) later [4] Regarding the body site distribution of BCCs, we found a prevalence of BCCs on the scalp, and upper extremities in females (i.e. patient number 1, 13, 16) whereas the back (lower portion) and lower extremities were preferentially involved in males (i.e. patient number 3, 4, 7). The face did not show any difference in term of BCCs distribution. According with previous data [24], the involvement of the scalp was typically found in females; however, a different distribution was found for the back and the extremities. In fact, Winnies et al found a prevalence of BCCs on the lower extremities and back in females. This could simply be related to a higher number of patients included in their study compared to our population.

No firm evidence for a genotype/phenotype correlation in NBCCS has been demonstrated, and there is adequate variation in single families to believe that environmental exposure, and perhaps modifier genes, may justify much of the variation. The presence or absence of a *PTCH1* mutation, mutation type, and gender have virtually no impact on the onset age of the first BCC or on the numbers of BCCs developed by the patient. This is clinically relevant as the finding of a specific *PTCH1* mutation does not appear to give prognostic information about the likely age of onset of BCC or the clinical disease burden. In NBCC Syndrome patients, neither the age of onset of BCCs or the number of BCCs that develop can be predicted by the presence or absence of a *PTCH1* mutation or by the type of the mutation [25].

A review of the literature highlights that AML was rarely associated to NBCCS because it was reported in only a few patients, confirming that an advanced age may be at a greater risk of presenting AML in NBCCS [26]. In disagreement with this reports in our case series two of 18 (11%) patients were affected by AML (patient number 8 and 14); therefore this tumor could represent not only an incidental finding but a potential sign of an hereditary disorder, that might be as useful as KCOTs as a screening criterion for the identification of *PTCH1* gene-carrier individuals at risk of NBCCS.

AMLs arise from remnants of dental lamina, an embryonic structure that normally differentiates into tooth buds and enamel-producing cells during odontogenesis. The dysregulation of the PTCH1-SHH signaling pathway in the interactions between epithelium and mesenchyme may induce the formation of the KCOTs and AMLs.

NBCCS associated Keratocysts and AMLs presumably arise from precursor cells that contain a hereditary “first hit”, and the allelic loss represents the loss of the normal allele [27], [28]. Sporadic KCOTs and AMLs may arise from susceptible cells in which two of the somatic “hits” have occurred, one of them manifesting as allelic loss. The *PTCH1* gene may function as a “gatekeeper gene” [29]. Indeed, this model has been proposed whereby inactivation of a gatekeeper gene is required for passing the genetic threshold of the neoplastic process in a given tissue. It is possible that cells of the KCOTs and AMLs, after losing the *PTCH1* function, become targets of other genetic alterations.

The dermatologic features of NBCCS have a significant impact on affected individuals. Among NBCCs patients presented in this study the number of BCCs varies from a few to several thousands, ranges in size from 1 to 30 mm and includes two or more histologic sub-types of superficial, nodular, pigmented patterns and fibroepithelioma of Pinkus. This last peculiar dermatological lesion was recently described by *Go JW et al.* as one of the several sub-types of cutaneous BCCs associated with the NBCCS tumor spectrum [30].

In the current study, molecular characterization showed a germline *PTCH1* mutations in 9 patients [Table 1].

The geno-phenotype characterization in the presented NBCCS cases shows no correlation between NBCCS specific clinical features and particular functional domains inside this gene, together with an ample phenotypic variability. This includes cases both with a marked familial cancer aggregation and apparently sporadic, in which the association of KCOTs and BCCs is present only in single individuals [Table 2]. On the other hand, it seems rather unclear how, under the same mutation and family history, a NBCCS phenotype only appears in some cases: for instance, the same mutation associated to NBCCS phenotype in a patient does not match with such phenotype in other carriers of the same mutation, either in the same family [Figure 2] or in other NBCCS correlated families.

At this regard, we recently reported the missense mutation c.3277G**>**C (p.G1093R) in exon 19 of the *PTCH1* gene – which had been previously reported only in non-syndromic KCOTs - in a familiar case (father and daughter of patient number 1) of classic NBCCS phenotype [17]. This observation suggests that this missense mutation might take part in the pathogenesis of NBCCS as well as in a subset of non syndromic KCOTs. These evidences of the wide inter- and intra-familial phenotypic variability could be due to lower penetrance of *PTCH1* gene and is likely to result from the action of modifier genes. There is evidence that the manifestations of many genetic disorders are influenced by so-called ‘modifying’ genes distinct from the disease locus. Anyway, the final NBCCS phenotypic outcome might be also modified by epigenetic factors such as ethnicity and environmental factors: among these the ultraviolet (UV) light is one of the high-risk factors for BCC development in NBCCS.

Overall, the multiple and early-onset KCOTs characteristic of NBCCS can serve as premonitory physical stigmata for an underlying cancer predisposition: in our opinion, every patient with a KCOTs (or AMLs) should be further evaluated and, if necessary, put under strict cancer surveillance. In conclusion, the current findings indicate a possible strategy that may be followed for the identification and management of NBCCS patients for which the early diagnosis of clinical manifestations is crucial because of the risk of medulloblastoma, ovarian fibroma and other neoplasms of the NBCCS tumor spectrum.

## Materials and Methods

### Patients and Tumor Samples

From 1994 to 2011, all keratocystic odontogenic tumors (embedded in paraffin) belonging to 70 patients were selected through the examination of the archives of the Pathology Departments of the Universities of Modena and Napoli.

The hematoxylin and eosin-stained slides were re-evaluated to confirm the diagnosis according to WHO classification of 1992 [10]. If the final diagnosis could not be made through histopathologic evaluation alone, clinical and radiographic findings were considered. When the original slides were not representative or were inadequate, new additional slides were prepared from paraffin blocks. In case of controversies, another oral pathologist was consulted in order to ensure that the final diagnosis was correct.

For purposes of recruitment for this study, criteria used for diagnosis of NBCC syndrome were the presence of 2 major, or one major and 2 minor criteria. The major criteria included multiple BCCs or one BCC before 30 years, keratocysts of the jaw, palmar/plantar pits and lamellar calcification of the falx cerebri on skull radiograph. Minor criteria included spina bifida occulta or other vertebral anomalies, brachymetacarpaly in at least one limb, hypertelorism or telecanthus, frontal bossing, rib anomalies (bifid, synostosed, hypoplastic), ovarian fibroma, medulloblastoma, flame shaped lucencies in the phalanges, and brachymetacarpaly in the 4 limbs. One diagnosis was also established by the presence of a first degree relative with NBCC and one major or two minor criteria. [4]. The anatomic distribution of BCCS was recorded according to six body sites (head and neck, M-face, Scalp, upper extremities, lower extremities, trunk). All patients were screened for the presence of BCCs by using a combined clinical-dermoscopic exam [31].

### Family History

Detailed family histories were collected for each patient by interviewing the patients and/or their relatives. Verification of cancer occurrence among family members could be obtained in the majority of patients through clinical charts, pathologic records, or death certificates. Through the reconstruction of the genealogic tree, we identified 18 patients with NBCCS.

Mann-Whitney Test (α<0.05) was applied to assess the probability of KCOTs per patients and and KCOTs onset year, respectively, in NBCCS patients versus non syndromic patients. Statistical analysis was performed using GraphPad 5.0 (Prism 5.0, GraphPad Software, Inc., San Diego, CA).

### Mutational Analysis

The peripheral blood samples were collected from the NBCCS proband and their first-degree relatives. Written informed consent, agreeing to peripheral blood sampling and genetic analysis, was obtained from each patient enrolled in the study. Molecular analysis of *PTCH1* was performed as previously described [26]. The *PTCH1* cDNA sequence from GenBank (Accession number U59464.1) was used as a reference sequence, where the A of the ATG translation initiation start site represents nucleotide +1.

An Institutional Review Board (IRB) approval was obtained and the study was conducted according to the Declaration of Helsinki Principles. All patients provided their written informed consent for the management of personal data and for publication of their photographs before participating into the study.

A council of senior specialists at the same University Department (Prof. Alberto Giannetti, Prof. Anto De Pol and Prof. Cristina Magnoni) reviewed and approved the study design, inclusion/exclusion criteria.
